# Complete chloroplast genome sequence of *Swertia mileensis* (Gentianaceae): a medicinal species endemic to China

**DOI:** 10.1080/23802359.2019.1693303

**Published:** 2019-11-20

**Authors:** Cong-Wei Yang, Ying-Ying Liu, Ying-Min Zhang, Xiao-Hui Ma, Zi-Gang Qian, Guo-Dong Li

**Affiliations:** a Faculty of Traditional Chinese Pharmacy, Yunnan University of Chinese Medicine, Kunming, China;; b Yunnan Insitute for Food and Drug, Kunming, China;; c Yunnan Key Laboratory for Dai and Yi Medicines, Yunnan University of Chinese Medicine, Kunming, China

**Keywords:** *Swertia mileensis*, chloroplast genome, medicinal plant, phylogenomic analysis

## Abstract

*Swertia mileensis* is an important medicinal plant endemic to South-east Yunnan, China, which has been widely used to treat icteric hepatitis. The complete chloroplast genome sequence of *S. mileensis* is presented in this study, the total size is 153,015 bp in length with a typical quadripartite structure including a pair of inverted repeat (IRs, 25,786 bp) regions separated by a large single copy (LSC, 83,048 bp) region and a small single copy (SSC, 18,395 bp) region. The overall GC content of it is 38.2%. The cp genome has 134 annotated genes, including 85 protein-coding genes, 37 tRNA genes and 8 rRNA genes. Among these genes, nine genes have one intron and two genes contain two introns. The phylogenetic tree based on 16 complete plastomes of support close relationships among two species of *Swertia* with 100% bootstrap value.

*Swertia mileensis* is a perennial herb of the family Gentianaceae and restricted to narrow distribution in a wide karst valley basin of eastern Yunnan province (Liu et al. 2018). Known as “Qingyedan”, it is used as a traditional Chinese medicine which can nourish liver qi for promoting bile flow and usually used to treat icteric hepatitis (Chinese Pharmacopoeia Commission 2015). The natural resources of *S. mileensis* have rapidly decreased over recent years because of growing demand of the market, overexploitation and habitat destruction (Huang et al. 2016). In order to protect decreasing natural genetic resources of *S. mileensis*, in this study we assembled and characterized the complete chloroplast genome of *S. mileensis,* it will provide potential genetic resource for further conservation and management strategies.

The fresh leaves of *S. mileensis* were collected from Mengzi Country (24°11′N, 103°26′E), Yunnan province and voucher specimens (5325260426) were deposited in Herbarium of Yunnan University of Chinese Medicine. The total genomic DNA was extracted using plant DNA (Bioteke Corporation, China) and sequencing was performed on an Illumina HiSeq 2500 platform (Illumina Inc., SanDiego, CA). The filtered reads were assembled using NOVOPlasty (Dierckxsens et al. 2017) with complete genome of its close relative *S. mussotii* (Xiang et al. 2016) as the reference. The assembled chloroplast genome was annotated with the online annotation tool GeSeq (Tillich et al. 2017), and the annotation was corrected using Geneious R11 11.1.5 (Biomatters Ltd., Auckland, New Zealand).

The complete chloroplast genome of *S. mileensis* was a quadripartite and 153,015 bp in length (GenBank accession No.: MN609998), containing a large single copy (LSC) region of 83,048 bp and a small single copy (SSC) region of 18,395 bp, separated by a pair of inverted repeat (IRs) regions of 25,786 bp. The cp genome possesses 134 annotated genes, including 85 protein-coding genes, 37 tRNA genes and 8 rRNA genes. Among all of these genes, four rRNA genes (4.5, 5, 16 and 23 rRNA), seven protein-coding genes (*ndhB*, *rpl2*, *rpl23*, *rps7*, *rps12*, *ycf2* and *ycf15*), and seven tRNA genes (*trnA-UGC*, *trnl-CAU*, *trnl-GAU*, *trnL-CAA*, *trnN-GUU*, *trnR-ACG*, *trnV-GAC*) occur in double copies. The overall GC-content of the whole plastome is 38.2%, while the corresponding values of the LSC, SSC, and IR regions are 36.3%, 31.9%, and 43.3%, respectively.

To determine the phylogenetic position of *S. mileensis*, a total of 16 species used to construct the phylogenetic tree among the most of Gentianales species with Gentianaceae, Apocynaceae, Rubiaceae, and two Solanales species as outgroups. All of the plastomes were aligned using MAFFT v.7 (Katoh and Standley 2013), and the RAxML (Stamatakis et al. 2014) inference was performed by using GTR model with support for branches evaluated by 1000 bootstrap replicates (Figure 1). The phylogenetic tree shown that *S. mileensis* is nested in monophyletic clade of tribe Gentianaceae, and closely related with *S. mussotii*. The complete chloroplast genome of *S. mileensis* will provide a useful resource for the conservation genetics of this species as well as for the phylogenetic studies of Gentianaceae.

**Figure 1.**
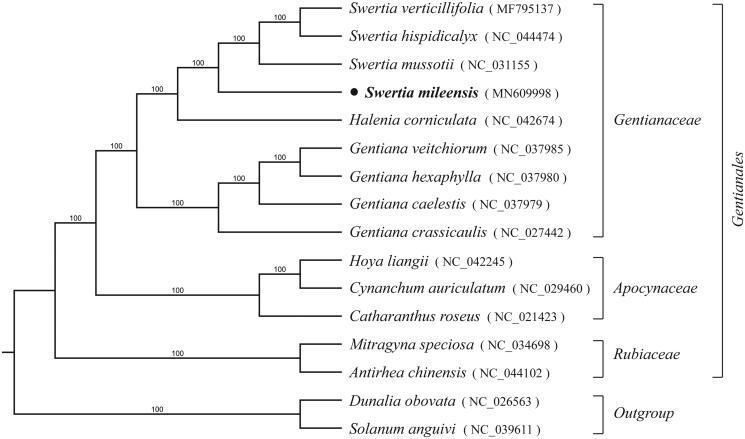
Maximum likelihood phylogenetic tree inferred from 16 chloroplast genomes. Bootstrap support values >50% are indicated next to the branches.
